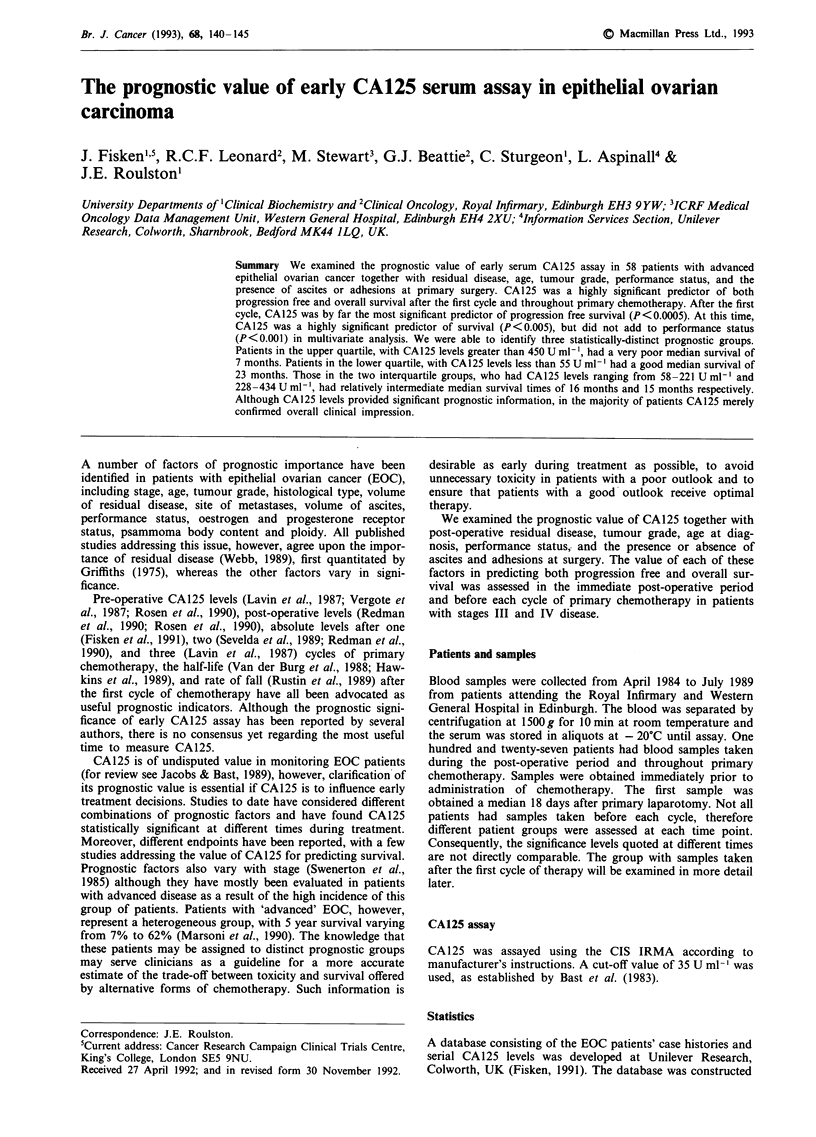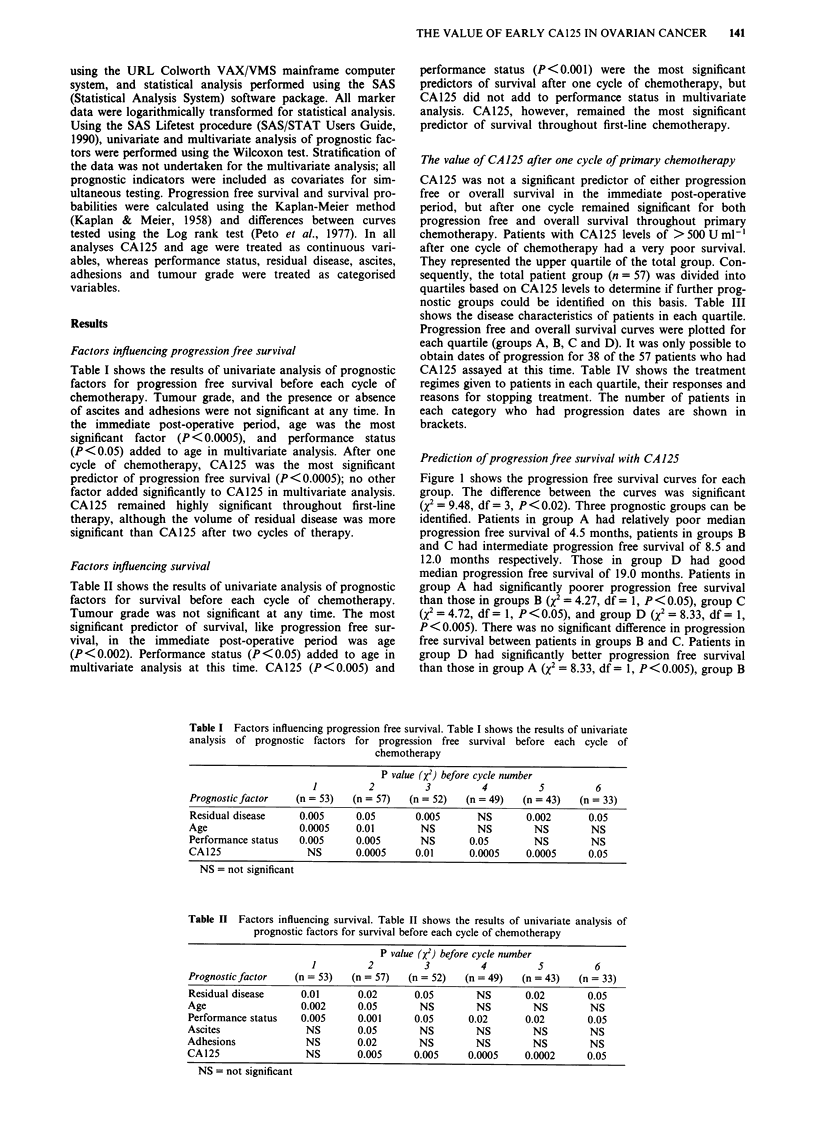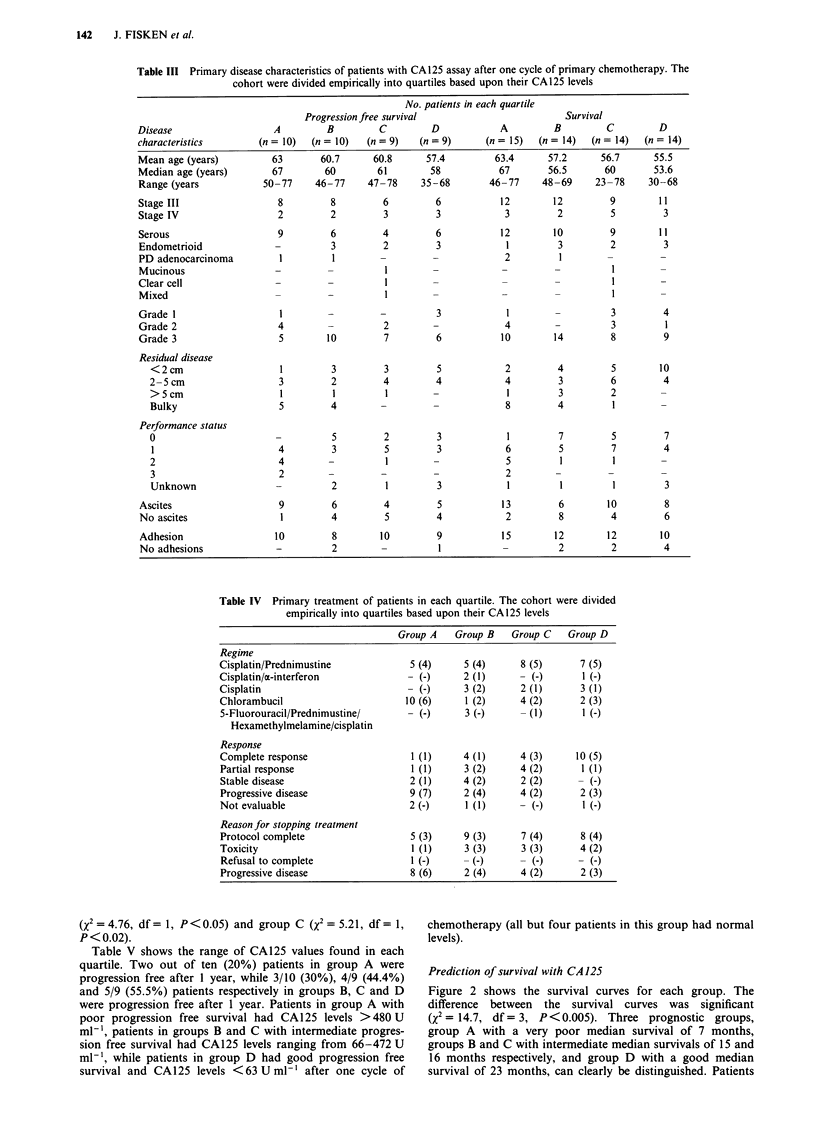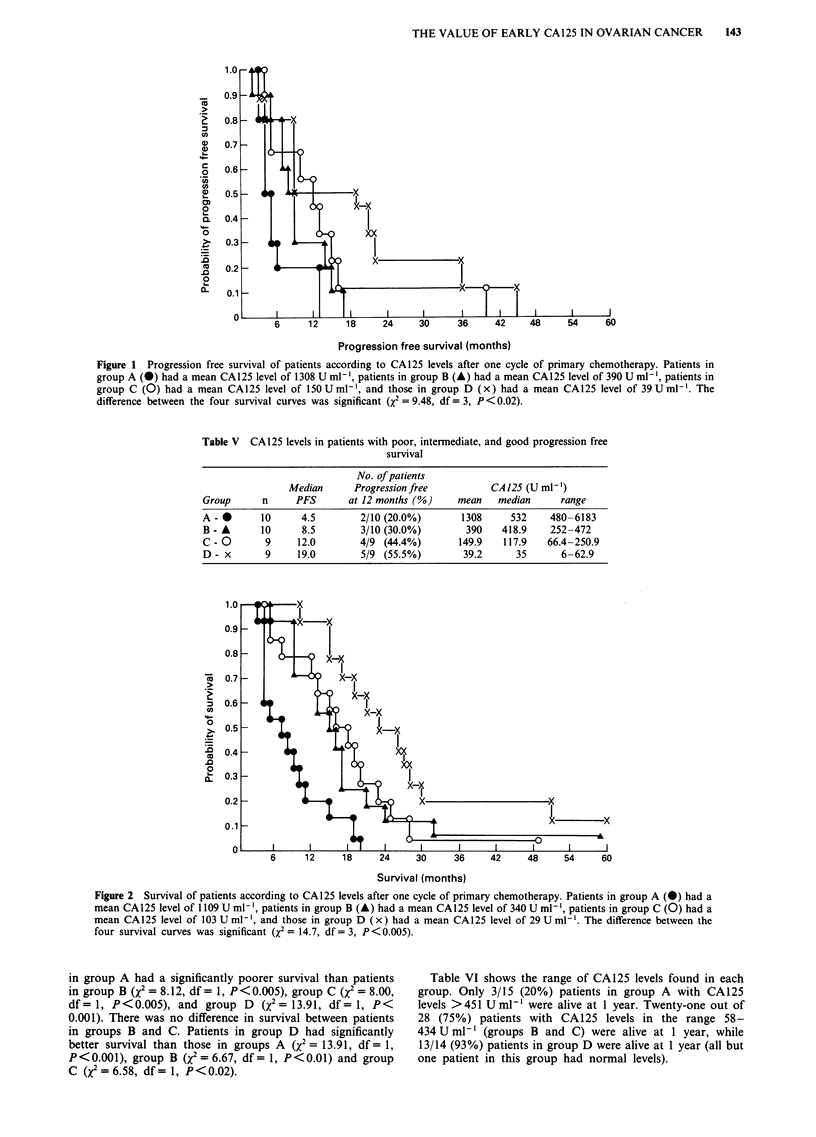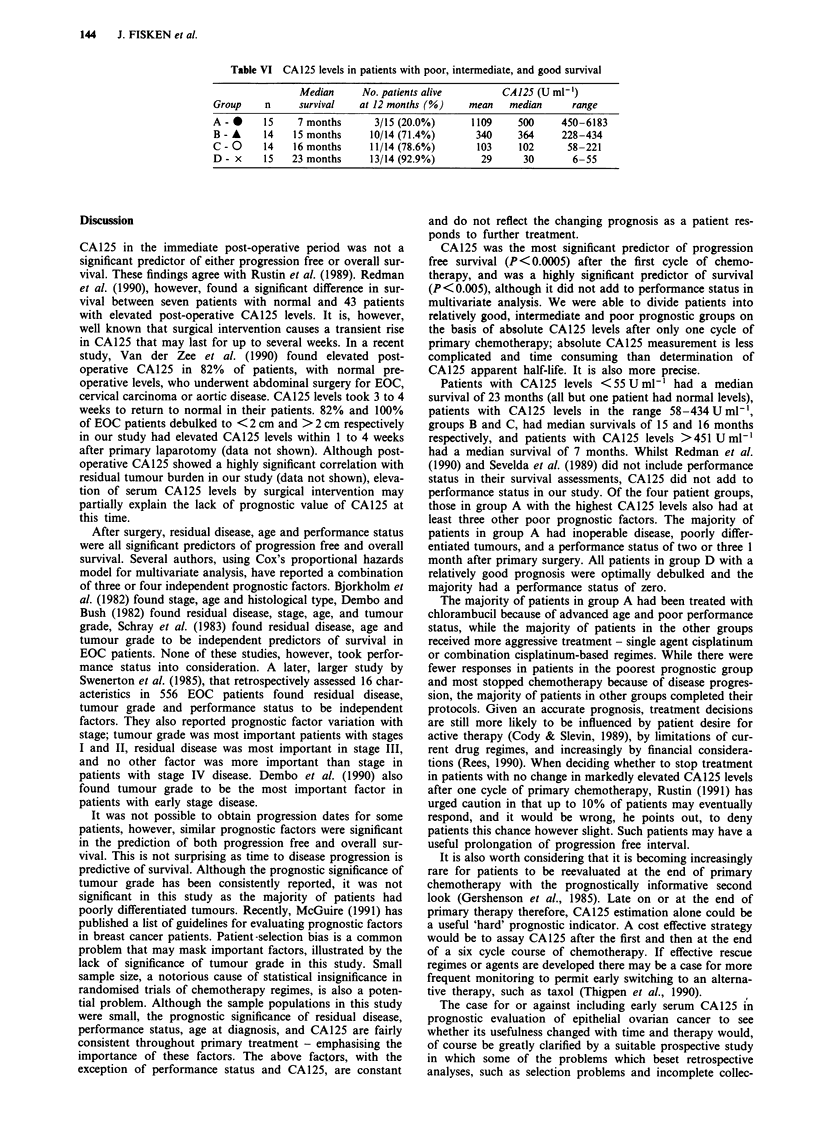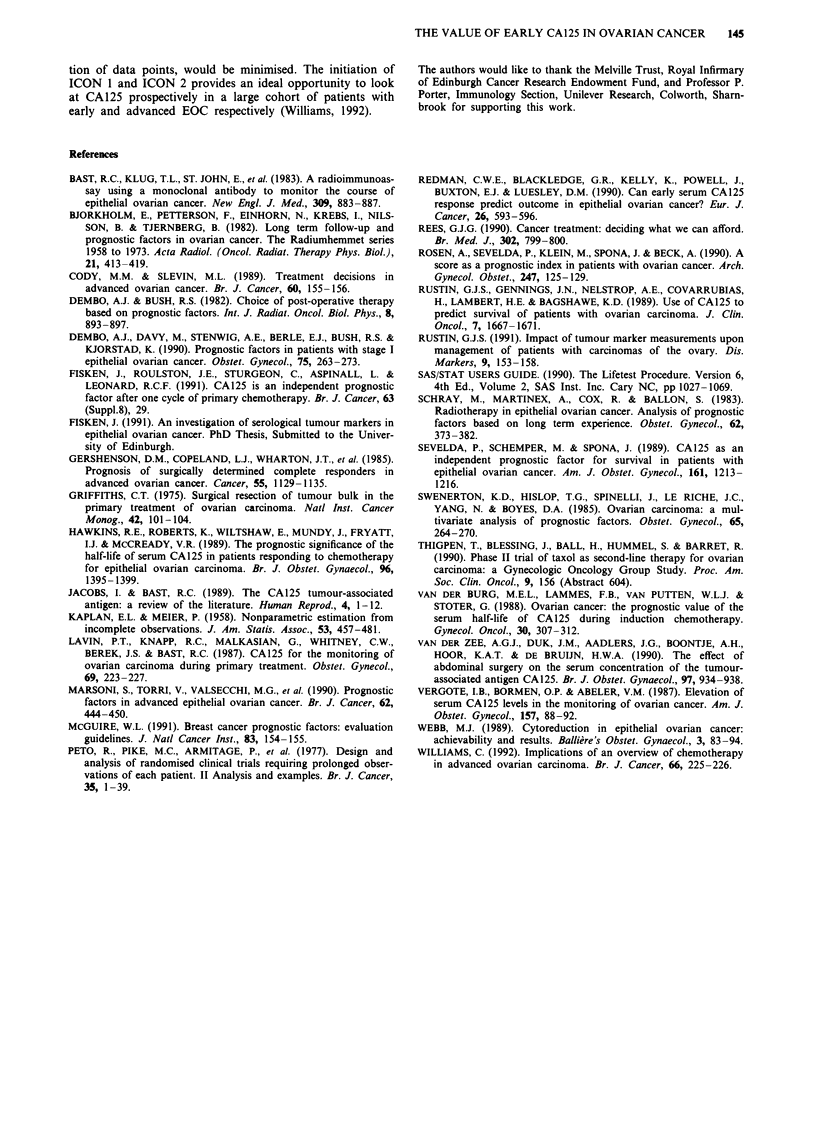# The prognostic value of early CA125 serum assay in epithelial ovarian carcinoma.

**DOI:** 10.1038/bjc.1993.302

**Published:** 1993-07

**Authors:** J. Fisken, R. C. Leonard, M. Stewart, G. J. Beattie, C. Sturgeon, L. Aspinall, J. E. Roulston

**Affiliations:** University Department of Clinical Biochemistry, Royal Infirmary, Edinburgh, UK.

## Abstract

We examined the prognostic value of early serum CA125 assay in 58 patients with advanced epithelial ovarian cancer together with residual disease, age, tumour grade, performance status, and the presence of ascites or adhesions at primary surgery. CA125 was a highly significant predictor of both progression free and overall survival after the first cycle and throughout primary chemotherapy. After the first cycle, CA125 was by far the most significant predictor of progression free survival (P < 0.0005). At this time, CA125 was a highly significant predictor of survival (P < 0.005), but did not add to performance status (P < 0.001) in multivariate analysis. We were able to identify three statistically-distinct prognostic groups. Patients in the upper quartile, with CA125 levels greater than 450 U ml-1, had a very poor median survival of 7 months. Patients in the lower quartile, with CA125 levels less than 55 U ml-1 had a good median survival of 23 months. Those in the two interquartile groups, who had CA125 levels ranging from 58-221 U ml-1 and 228-434 U ml-1, had relatively intermediate median survival times of 16 months and 15 months respectively. Although CA125 levels provided significant prognostic information, in the majority of patients CA125 merely confirmed overall clinical impression.


 © Macmillan Press Ltd., 1993

### The prognostic value of early CA125 serum assay in epithelial ovarian carcinoma

J. Fisken[1,5], R.C.F. Leonard[2], M. Stewart[3], G.J. Beattie[2], C. Sturgeon[1], L. Aspinall[4] & J.E. Roulston[1]

*University Departments of [1]Clinical Biochemistry and [2]Clinical Oncology, Royal Infirmary, Edinburgh EH3 9YW; [3]ICRF Medical Oncology Data Management Unit, Western General Hospital, Edinburgh EH4 2XU; [4]Information Services Section, Unilever Research, Colworth, Sharnbrook, Bedford MK44 1LQ, UK.*

**Summary** We examined the prognostic value of early serum CA125 assay in 58 patients with advanced epithelial ovarian cancer together with residual disease, age, tumour grade, performance status, and the presence of ascites or adhesions at primary surgery. CA125 was a highly significant predictor of both progression free and overall survival after the first cycle and throughout primary chemotherapy. After the first cycle, CA125 was by far the most significant predictor of progression free survival ($P<0.0005$). At this time, CA125 was a highly significant predictor of survival ($P<0.005$), but did not add to performance status ($P<0.001$) in multivariate analysis. We were able to identify three statistically-distinct prognostic groups. Patients in the upper quartile, with CA125 levels greater than 450 U ml$^{-1}$, had a very poor median survival of 7 months. Patients in the lower quartile, with CA125 levels less than 55 U ml$^{-1}$ had a good median survival of 23 months. Those in the two interquartile groups, who had CA125 levels ranging from 58–221 U ml$^{-1}$ and 228–434 U ml$^{-1}$, had relatively intermediate median survival times of 16 months and 15 months respectively. Although CA125 levels provided significant prognostic information, in the majority of patients CA125 merely confirmed overall clinical impression.

A number of factors of prognostic importance have been identified in patients with epithelial ovarian cancer (EOC), including stage, age, tumour grade, histological type, volume of residual disease, site of metastases, volume of ascites, performance status, oestrogen and progesterone receptor status, psammoma body content and ploidy. All published studies addressing this issue, however, agree upon the importance of residual disease (Webb, 1989), first quantitated by Griffiths (1975), whereas the other factors vary in significance.

Pre-operative CA125 levels (Lavin *et al.*, 1987; Vergote *et al.*, 1987; Rosen *et al.*, 1990), post-operative levels (Redman *et al.*, 1990; Rosen *et al.*, 1990), absolute levels after one (Fisken *et al.*, 1991), two (Sevelda *et al.*, 1989; Redman *et al.*, 1990), and three (Lavin *et al.*, 1987) cycles of primary chemotherapy, the half-life (Van der Burg *et al.*, 1988; Hawkins *et al.*, 1989), and rate of fall (Rustin *et al.*, 1989) after the first cycle of chemotherapy have all been advocated as useful prognostic indicators. Although the prognostic significance of early CA125 assay has been reported by several authors, there is no consensus yet regarding the most useful time to measure CA125.

CA125 is of undisputed value in monitoring EOC patients (for review see Jacobs & Bast, 1989), however, clarification of its prognostic value is essential if CA125 is to influence early treatment decisions. Studies to date have considered different combinations of prognostic factors and have found CA125 statistically significant at different times during treatment. Moreover, different endpoints have been reported, with a few studies addressing the value of CA125 for predicting survival. Prognostic factors also vary with stage (Swenerton *et al.*, 1985) although they have mostly been evaluated in patients with advanced disease as a result of the high incidence of this group of patients. Patients with 'advanced' EOC, however, represent a heterogeneous group, with 5 year survival varying from 7% to 62% (Marsoni *et al.*, 1990). The knowledge that these patients may be assigned to distinct prognostic groups may serve clinicians as a guideline for a more accurate estimate of the trade-off between toxicity and survival offered by alternative forms of chemotherapy. Such information is desirable as early during treatment as possible, to avoid unnecessary toxicity in patients with a poor outlook and to ensure that patients with a good outlook receive optimal therapy.

We examined the prognostic value of CA125 together with post-operative residual disease, tumour grade, age at diagnosis, performance status, and the presence or absence of ascites and adhesions at surgery. The value of each of these factors in predicting both progression free and overall survival was assessed in the immediate post-operative period and before each cycle of primary chemotherapy in patients with stages III and IV disease.

Correspondence: J.E. Roulston.
[5]Current address: Cancer Research Campaign Clinical Trials Centre, King's College, London SE5 9NU.
Received 27 April 1992; and in revised form 30 November 1992.

### Patients and samples

Blood samples were collected from April 1984 to July 1989 from patients attending the Royal Infirmary and Western General Hospital in Edinburgh. The blood was separated by centrifugation at 1500 *g* for 10 min at room temperature and the serum was stored in aliquots at − 20°C until assay. One hundred and twenty-seven patients had blood samples taken during the post-operative period and throughout primary chemotherapy. Samples were obtained immediately prior to administration of chemotherapy. The first sample was obtained a median 18 days after primary laparotomy. Not all patients had samples taken before each cycle, therefore different patient groups were assessed at each time point. Consequently, the significance levels quoted at different times are not directly comparable. The group with samples taken after the first cycle of therapy will be examined in more detail later.

### CA125 assay

CA125 was assayed using the CIS IRMA according to manufacturer's instructions. A cut-off value of 35 U ml$^{-1}$ was used, as established by Bast *et al.* (1983).

### Statistics

A database consisting of the EOC patients' case histories and serial CA125 levels was developed at Unilever Research, Colworth, UK (Fisken, 1991). The database was constructed using the URL Colworth VAX/VMS mainframe computer system, and statistical analysis performed using the SAS (Statistical Analysis System) software package. All marker data were logarithmically transformed for statistical analysis. Using the SAS Lifetest procedure (SAS/STAT Users Guide, 1990), univariate and multivariate analysis of prognostic factors were performed using the Wilcoxon test. Stratification of the data was not undertaken for the multivariate analysis; all prognostic indicators were included as covariates for simultaneous testing. Progression free survival and survival probabilities were calculated using the Kaplan-Meier method (Kaplan & Meier, 1958) and differences between curves tested using the Log rank test (Peto *et al.*, 1977). In all analyses CA125 and age were treated as continuous variables, whereas performance status, residual disease, ascites, adhesions and tumour grade were treated as categorised variables.

### Results

### *Factors influencing progression free survival*

Table I shows the results of univariate analysis of prognostic factors for progression free survival before each cycle of chemotherapy. Tumour grade, and the presence or absence of ascites and adhesions were not significant at any time. In the immediate post-operative period, age was the most significant factor ($P<0.0005$), and performance status ($P<0.05$) added to age in multivariate analysis. After one cycle of chemotherapy, CA125 was the most significant predictor of progression free survival ($P<0.0005$); no other factor added significantly to CA125 in multivariate analysis. CA125 remained highly significant throughout first-line therapy, although the volume of residual disease was more significant than CA125 after two cycles of therapy.

### *Factors influencing survival*

Table II shows the results of univariate analysis of prognostic factors for survival before each cycle of chemotherapy. Tumour grade was not significant at any time. The most significant predictor of survival, like progression free survival, in the immediate post-operative period was age ($P<0.002$). Performance status ($P<0.05$) added to age in multivariate analysis at this time. CA125 ($P<0.005$) and performance status ($P<0.001$) were the most significant predictors of survival after one cycle of chemotherapy, but CA125 did not add to performance status in multivariate analysis. CA125, however, remained the most significant predictor of survival throughout first-line chemotherapy.

### *The value of CA125 after one cycle of primary chemotherapy*

CA125 was not a significant predictor of either progression free or overall survival in the immediate post-operative period, but after one cycle remained significant for both progression free and overall survival throughout primary chemotherapy. Patients with CA125 levels of $>500\ \mathrm{U\ ml^{-1}}$ after one cycle of chemotherapy had a very poor survival. They represented the upper quartile of the total group. Consequently, the total patient group ($n = 57$) was divided into quartiles based on CA125 levels to determine if further prognostic groups could be identified on this basis. Table III shows the disease characteristics of patients in each quartile. Progression free and overall survival curves were plotted for each quartile (groups A, B, C and D). It was only possible to obtain dates of progression for 38 of the 57 patients who had CA125 assayed at this time. Table IV shows the treatment regimes given to patients in each quartile, their responses and reasons for stopping treatment. The number of patients in each category who had progression dates are shown in brackets.

### *Prediction of progression free survival with CA125*

Figure 1 shows the progression free survival curves for each group. The difference between the curves was significant ($\chi^2 = 9.48$, df = 3, $P<0.02$). Three prognostic groups can be identified. Patients in group A had relatively poor median progression free survival of 4.5 months, patients in groups B and C had intermediate progression free survival of 8.5 and 12.0 months respectively. Those in group D had good median progression free survival of 19.0 months. Patients in group A had significantly poorer progression free survival than those in groups B ($\chi^2 = 4.27$, df = 1, $P<0.05$), group C ($\chi^2 = 4.72$, df = 1, $P<0.05$), and group D ($\chi^2 = 8.33$, df = 1, $P<0.005$). There was no significant difference in progression free survival between patients in groups B and C. Patients in group D had significantly better progression free survival than those in group A ($\chi^2 = 8.33$, df = 1, $P<0.005$), group B

**Table I** Factors influencing progression free survival. Table I shows the results of univariate analysis of prognostic factors for progression free survival before each cycle of chemotherapy

| | P *value* ($\chi^2$) *before cycle number* | | | | | |
|---|---|---|---|---|---|---|
| *Prognostic factor* | *1* (n = 53) | *2* (n = 57) | *3* (n = 52) | *4* (n = 49) | *5* (n = 43) | *6* (n = 33) |
| Residual disease | 0.005 | 0.05 | 0.005 | NS | 0.002 | 0.05 |
| Age | 0.0005 | 0.01 | NS | NS | NS | NS |
| Performance status | 0.005 | 0.005 | NS | 0.05 | NS | NS |
| CA125 | NS | 0.0005 | 0.01 | 0.0005 | 0.0005 | 0.05 |

NS = not significant

**Table II** Factors influencing survival. Table II shows the results of univariate analysis of prognostic factors for survival before each cycle of chemotherapy

| | P *value* ($\chi^2$) *before cycle number* | | | | | |
|---|---|---|---|---|---|---|
| *Prognostic factor* | *1* (n = 53) | *2* (n = 57) | *3* (n = 52) | *4* (n = 49) | *5* (n = 43) | *6* (n = 33) |
| Residual disease | 0.01 | 0.02 | 0.05 | NS | 0.02 | 0.05 |
| Age | 0.002 | 0.05 | NS | NS | NS | NS |
| Performance status | 0.005 | 0.001 | 0.05 | 0.02 | 0.02 | 0.05 |
| Ascites | NS | 0.05 | NS | NS | NS | NS |
| Adhesions | NS | 0.02 | NS | NS | NS | NS |
| CA125 | NS | 0.005 | 0.005 | 0.0005 | 0.0002 | 0.05 |

NS = not significant

**Table III** Primary disease characteristics of patients with CA125 assay after one cycle of primary chemotherapy. The cohort were divided empirically into quartiles based upon their CA125 levels

| | *No. patients in each quartile* | | | | | | | |
|---|---|---|---|---|---|---|---|---|
| | *Progression free survival* | | | | *Survival* | | | |
| *Disease characteristics* | *A* (*n* = 10) | *B* (*n* = 10) | *C* (*n* = 9) | *D* (*n* = 9) | A (*n* = 15) | *B* (*n* = 14) | *C* (*n* = 14) | *D* (*n* = 14) |
| Mean age (years) | 63 | 60.7 | 60.8 | 57.4 | 63.4 | 57.2 | 56.7 | 55.5 |
| Median age (years) | 67 | 60 | 61 | 58 | 67 | 56.5 | 60 | 53.6 |
| Range (years | 50–77 | 46–77 | 47–78 | 35–68 | 46–77 | 48–69 | 23–78 | 30–68 |
| Stage III | 8 | 8 | 6 | 6 | 12 | 12 | 9 | 11 |
| Stage IV | 2 | 2 | 3 | 3 | 3 | 2 | 5 | 3 |
| Serous | 9 | 6 | 4 | 6 | 12 | 10 | 9 | 11 |
| Endometrioid | – | 3 | 2 | 3 | 1 | 3 | 2 | 3 |
| PD adenocarcinoma | 1 | 1 | – | – | 2 | 1 | – | – |
| Mucinous | – | – | 1 | – | – | – | 1 | – |
| Clear cell | – | – | 1 | – | – | – | 1 | – |
| Mixed | – | – | 1 | – | – | – | 1 | – |
| Grade 1 | 1 | – | – | 3 | 1 | – | 3 | 4 |
| Grade 2 | 4 | – | 2 | – | 4 | – | 3 | 1 |
| Grade 3 | 5 | 10 | 7 | 6 | 10 | 14 | 8 | 9 |
| *Residual disease* | | | | | | | | |
| <2 cm | 1 | 3 | 3 | 5 | 2 | 4 | 5 | 10 |
| 2–5 cm | 3 | 2 | 4 | 4 | 4 | 3 | 6 | 4 |
| >5 cm | 1 | 1 | 1 | – | 1 | 3 | 2 | – |
| Bulky | 5 | 4 | – | – | 8 | 4 | 1 | – |
| *Performance status* | | | | | | | | |
| 0 | – | 5 | 2 | 3 | 1 | 7 | 5 | 7 |
| 1 | 4 | 3 | 5 | 3 | 6 | 5 | 7 | 4 |
| 2 | 4 | – | 1 | – | 5 | 1 | 1 | – |
| 3 | 2 | – | – | – | 2 | – | – | – |
| Unknown | – | 2 | 1 | 3 | 1 | 1 | 1 | 3 |
| Ascites | 9 | 6 | 4 | 5 | 13 | 6 | 10 | 8 |
| No ascites | 1 | 4 | 5 | 4 | 2 | 8 | 4 | 6 |
| Adhesion | 10 | 8 | 10 | 9 | 15 | 12 | 12 | 10 |
| No adhesions | – | 2 | – | 1 | – | 2 | 2 | 4 |

**Table IV** Primary treatment of patients in each quartile. The cohort were divided empirically into quartiles based upon their CA125 levels

| | *Group A* | *Group B* | *Group C* | *Group D* |
|---|---|---|---|---|
| *Regime* | | | | |
| Cisplatin/Prednimustine | 5 (4) | 5 (4) | 8 (5) | 7 (5) |
| Cisplatin/α-interferon | – (-) | 2 (1) | – (-) | 1 (-) |
| Cisplatin | – (-) | 3 (2) | 2 (1) | 3 (1) |
| Chlorambucil | 10 (6) | 1 (2) | 4 (2) | 2 (3) |
| 5-Fluorouracil/Prednimustine/ Hexamethylmelamine/cisplatin | – (-) | 3 (-) | – (1) | 1 (-) |
| *Response* | | | | |
| Complete response | 1 (1) | 4 (1) | 4 (3) | 10 (5) |
| Partial response | 1 (1) | 3 (2) | 4 (2) | 1 (1) |
| Stable disease | 2 (1) | 4 (2) | 2 (2) | – (-) |
| Progressive disease | 9 (7) | 2 (4) | 4 (2) | 2 (3) |
| Not evaluable | 2 (-) | 1 (1) | – (-) | 1 (-) |
| *Reason for stopping treatment* | | | | |
| Protocol complete | 5 (3) | 9 (3) | 7 (4) | 8 (4) |
| Toxicity | 1 (1) | 3 (3) | 3 (3) | 4 (2) |
| Refusal to complete | 1 (-) | – (-) | – (-) | – (-) |
| Progressive disease | 8 (6) | 2 (4) | 4 (2) | 2 (3) |

($\chi^2 = 4.76$, df = 1, $P < 0.05$) and group C ($\chi^2 = 5.21$, df = 1, $P < 0.02$).

Table V shows the range of CA125 values found in each quartile. Two out of ten (20%) patients in group A were progression free after 1 year, while 3/10 (30%), 4/9 (44.4%) and 5/9 (55.5%) patients respectively in groups B, C and D were progression free after 1 year. Patients in group A with poor progression free survival had CA125 levels >480 U $ml^{-1}$, patients in groups B and C with intermediate progression free survival had CA125 levels ranging from 66–472 U $ml^{-1}$, while patients in group D had good progression free survival and CA125 levels <63 U $ml^{-1}$ after one cycle of chemotherapy (all but four patients in this group had normal levels).

### *Prediction of survival with CA125*

Figure 2 shows the survival curves for each group. The difference between the survival curves was significant ($\chi^2 = 14.7$, df = 3, $P < 0.005$). Three prognostic groups, group A with a very poor median survival of 7 months, groups B and C with intermediate median survivals of 15 and 16 months respectively, and group D with a good median survival of 23 months, can clearly be distinguished. Patients

**Figure 1** Progression free survival of patients according to CA125 levels after one cycle of primary chemotherapy. Patients in group A (●) had a mean CA125 level of 1308 U $ml^{-1}$, patients in group B (▲) had a mean CA125 level of 390 U $ml^{-1}$, patients in group C (○) had a mean CA125 level of 150 U $ml^{-1}$, and those in group D (×) had a mean CA125 level of 39 U $ml^{-1}$. The difference between the four survival curves was significant ($\chi^2 = 9.48$, df = 3, $P < 0.02$).

**Table V** CA125 levels in patients with poor, intermediate, and good progression free survival

| *Group* | n | *Median PFS* | *No. of patients Progression free at 12 months (%)* | *CA125* (U $ml^{-1}$) *mean* | *median* | *range* |
|---|---|---|---|---|---|---|
| A - ● | 10 | 4.5 | 2/10 (20.0%) | 1308 | 532 | 480–6183 |
| B - ▲ | 10 | 8.5 | 3/10 (30.0%) | 390 | 418.9 | 252–472 |
| C - ○ | 9 | 12.0 | 4/9 (44.4%) | 149.9 | 117.9 | 66.4–250.9 |
| D - × | 9 | 19.0 | 5/9 (55.5%) | 39.2 | 35 | 6–62.9 |

**Figure 2** Survival of patients according to CA125 levels after one cycle of primary chemotherapy. Patients in group A (●) had a mean CA125 level of 1109 U $ml^{-1}$, patients in group B (▲) had a mean CA125 level of 340 U $ml^{-1}$, patients in group C (○) had a mean CA125 level of 103 U $ml^{-1}$, and those in group D (×) had a mean CA125 level of 29 U $ml^{-1}$. The difference between the four survival curves was significant ($\chi^2 = 14.7$, df = 3, $P < 0.005$).

in group A had a significantly poorer survival than patients in group B ($\chi^2 = 8.12$, df = 1, $P < 0.005$), group C ($\chi^2 = 8.00$, df = 1, $P < 0.005$), and group D ($\chi^2 = 13.91$, df = 1, $P < 0.001$). There was no difference in survival between patients in groups B and C. Patients in group D had significantly better survival than those in groups A ($\chi^2 = 13.91$, df = 1, $P < 0.001$), group B ($\chi^2 = 6.67$, df = 1, $P < 0.01$) and group C ($\chi^2 = 6.58$, df = 1, $P < 0.02$).

Table VI shows the range of CA125 levels found in each group. Only 3/15 (20%) patients in group A with CA125 levels > 451 U $ml^{-1}$ were alive at 1 year. Twenty-one out of 28 (75%) patients with CA125 levels in the range 58–434 U $ml^{-1}$ (groups B and C) were alive at 1 year, while 13/14 (93%) patients in group D were alive at 1 year (all but one patient in this group had normal levels).

**Table VI** CA125 levels in patients with poor, intermediate, and good survival

| Group | n | Median survival | No. patients alive at 12 months (%) | CA125 ($U ml^{-1}$) mean | median | range |
|---|---|---|---|---|---|---|
| A - ● | 15 | 7 months | 3/15 (20.0%) | 1109 | 500 | 450-6183 |
| B - ▲ | 14 | 15 months | 10/14 (71.4%) | 340 | 364 | 228-434 |
| C - ○ | 14 | 16 months | 11/14 (78.6%) | 103 | 102 | 58-221 |
| D - × | 15 | 23 months | 13/14 (92.9%) | 29 | 30 | 6-55 |

### Discussion

CA125 in the immediate post-operative period was not a significant predictor of either progression free or overall survival. These findings agree with Rustin *et al.* (1989). Redman *et al.* (1990), however, found a significant difference in survival between seven patients with normal and 43 patients with elevated post-operative CA125 levels. It is, however, well known that surgical intervention causes a transient rise in CA125 that may last for up to several weeks. In a recent study, Van der Zee *et al.* (1990) found elevated post-operative CA125 in 82% of patients, with normal pre-operative levels, who underwent abdominal surgery for EOC, cervical carcinoma or aortic disease. CA125 levels took 3 to 4 weeks to return to normal in their patients. 82% and 100% of EOC patients debulked to $<2$ cm and $>2$ cm respectively in our study had elevated CA125 levels within 1 to 4 weeks after primary laparotomy (data not shown). Although post-operative CA125 showed a highly significant correlation with residual tumour burden in our study (data not shown), elevation of serum CA125 levels by surgical intervention may partially explain the lack of prognostic value of CA125 at this time.

After surgery, residual disease, age and performance status were all significant predictors of progression free and overall survival. Several authors, using Cox's proportional hazards model for multivariate analysis, have reported a combination of three or four independent prognostic factors. Bjorkholm *et al.* (1982) found stage, age and histological type, Dembo and Bush (1982) found residual disease, stage, age, and tumour grade, Schray *et al.* (1983) found residual disease, age and tumour grade to be independent predictors of survival in EOC patients. None of these studies, however, took performance status into consideration. A later, larger study by Swenerton *et al.* (1985), that retrospectively assessed 16 characteristics in 556 EOC patients found residual disease, tumour grade and performance status to be independent factors. They also reported prognostic factor variation with stage; tumour grade was most important patients with stages I and II, residual disease was most important in stage III, and no other factor was more important than stage in patients with stage IV disease. Dembo *et al.* (1990) also found tumour grade to be the most important factor in patients with early stage disease.

It was not possible to obtain progression dates for some patients, however, similar prognostic factors were significant in the prediction of both progression free and overall survival. This is not surprising as time to disease progression is predictive of survival. Although the prognostic significance of tumour grade has been consistently reported, it was not significant in this study as the majority of patients had poorly differentiated tumours. Recently, McGuire (1991) has published a list of guidelines for evaluating prognostic factors in breast cancer patients. Patient-selection bias is a common problem that may mask important factors, illustrated by the lack of significance of tumour grade in this study. Small sample size, a notorious cause of statistical insignificance in randomised trials of chemotherapy regimes, is also a potential problem. Although the sample populations in this study were small, the prognostic significance of residual disease, performance status, age at diagnosis, and CA125 are fairly consistent throughout primary treatment – emphasising the importance of these factors. The above factors, with the exception of performance status and CA125, are constant and do not reflect the changing prognosis as a patient responds to further treatment.

CA125 was the most significant predictor of progression free survival ($P<0.0005$) after the first cycle of chemotherapy, and was a highly significant predictor of survival ($P<0.005$), although it did not add to performance status in multivariate analysis. We were able to divide patients into relatively good, intermediate and poor prognostic groups on the basis of absolute CA125 levels after only one cycle of primary chemotherapy; absolute CA125 measurement is less complicated and time consuming than determination of CA125 apparent half-life. It is also more precise.

Patients with CA125 levels $<55\ U\ ml^{-1}$ had a median survival of 23 months (all but one patient had normal levels), patients with CA125 levels in the range $58-434\ U\ ml^{-1}$, groups B and C, had median survivals of 15 and 16 months respectively, and patients with CA125 levels $>451\ U\ ml^{-1}$ had a median survival of 7 months. Whilst Redman *et al.* (1990) and Sevelda *et al.* (1989) did not include performance status in their survival assessments, CA125 did not add to performance status in our study. Of the four patient groups, those in group A with the highest CA125 levels also had at least three other poor prognostic factors. The majority of patients in group A had inoperable disease, poorly differentiated tumours, and a performance status of two or three 1 month after primary surgery. All patients in group D with a relatively good prognosis were optimally debulked and the majority had a performance status of zero.

The majority of patients in group A had been treated with chlorambucil because of advanced age and poor performance status, while the majority of patients in the other groups received more aggressive treatment – single agent cisplatinum or combination cisplatinum-based regimes. While there were fewer responses in patients in the poorest prognostic group and most stopped chemotherapy because of disease progression, the majority of patients in other groups completed their protocols. Given an accurate prognosis, treatment decisions are still more likely to be influenced by patient desire for active therapy (Cody & Slevin, 1989), by limitations of current drug regimes, and increasingly by financial considerations (Rees, 1990). When deciding whether to stop treatment in patients with no change in markedly elevated CA125 levels after one cycle of primary chemotherapy, Rustin (1991) has urged caution in that up to 10% of patients may eventually respond, and it would be wrong, he points out, to deny patients this chance however slight. Such patients may have a useful prolongation of progression free interval.

It is also worth considering that it is becoming increasingly rare for patients to be reevaluated at the end of primary chemotherapy with the prognostically informative second look (Gershenson *et al.*, 1985). Late on or at the end of primary therapy therefore, CA125 estimation alone could be a useful 'hard' prognostic indicator. A cost effective strategy would be to assay CA125 after the first and then at the end of a six cycle course of chemotherapy. If effective rescue regimes or agents are developed there may be a case for more frequent monitoring to permit early switching to an alternative therapy, such as taxol (Thigpen *et al.*, 1990).

The case for or against including early serum CA125 in prognostic evaluation of epithelial ovarian cancer to see whether its usefulness changed with time and therapy would, of course be greatly clarified by a suitable prospective study in which some of the problems which beset retrospective analyses, such as selection problems and incomplete collection of data points, would be minimised. The initiation of ICON 1 and ICON 2 provides an ideal opportunity to look at CA125 prospectively in a large cohort of patients with early and advanced EOC respectively (Williams, 1992).

The authors would like to thank the Melville Trust, Royal Infirmary of Edinburgh Cancer Research Endowment Fund, and Professor P. Porter, Immunology Section, Unilever Research, Colworth, Sharnbrook for supporting this work.

### References

BAST, R.C., KLUG, T.L., ST. JOHN, E., *et al.* (1983). A radioimmunoassay using a monoclonal antibody to monitor the course of epithelial ovarian cancer. *New Engl. J. Med.*, **309**, 883–887.

BJORKHOLM, E., PETTERSON, F., EINHORN, N., KREBS, I., NILSSON, B. & TJERNBERG, B. (1982). Long term follow-up and prognostic factors in ovarian cancer. The Radiumhemmet series 1958 to 1973. *Acta Radiol. (Oncol. Radiat. Therapy Phys. Biol.)*, **21**, 413–419.

CODY, M.M. & SLEVIN, M.L. (1989). Treatment decisions in advanced ovarian cancer. *Br. J. Cancer*, **60**, 155–156.

DEMBO, A.J. & BUSH, R.S. (1982). Choice of post-operative therapy based on prognostic factors. *Int. J. Radiat. Oncol. Biol. Phys.*, **8**, 893–897.

DEMBO, A.J., DAVY, M., STENWIG, A.E., BERLE, E.J., BUSH, R.S. & KJORSTAD, K. (1990). Prognostic factors in patients with stage I epithelial ovarian cancer. *Obstet. Gynecol.*, **75**, 263–273.

FISKEN, J., ROULSTON, J.E., STURGEON, C., ASPINALL, L. & LEONARD, R.C.F. (1991). CA125 is an independent prognostic factor after one cycle of primary chemotherapy. *Br. J. Cancer*, **63** (Suppl.8), 29.

FISKEN, J. (1991). An investigation of serological tumour markers in epithelial ovarian cancer. PhD Thesis, Submitted to the University of Edinburgh.

GERSHENSON, D.M., COPELAND, L.J., WHARTON, J.T., *et al.* (1985). Prognosis of surgically determined complete responders in advanced ovarian cancer. *Cancer*, **55**, 1129–1135.

GRIFFITHS, C.T. (1975). Surgical resection of tumour bulk in the primary treatment of ovarian carcinoma. *Natl Inst. Cancer Monog.*, **42**, 101–104.

HAWKINS, R.E., ROBERTS, K., WILTSHAW, E., MUNDY, J., FRYATT, I.J. & MCCREADY, V.R. (1989). The prognostic significance of the half-life of serum CA125 in patients responding to chemotherapy for epithelial ovarian carcinoma. *Br. J. Obstet. Gynaecol.*, **96**, 1395–1399.

JACOBS, I. & BAST, R.C. (1989). The CA125 tumour-associated antigen: a review of the literature. *Human Reprod.*, **4**, 1–12.

KAPLAN, E.L. & MEIER, P. (1958). Nonparametric estimation from incomplete observations. *J. Am. Statis. Assoc.*, **53**, 457–481.

LAVIN, P.T., KNAPP, R.C., MALKASIAN, G., WHITNEY, C.W., BEREK, J.S. & BAST, R.C. (1987). CA125 for the monitoring of ovarian carcinoma during primary treatment. *Obstet. Gynecol.*, **69**, 223–227.

MARSONI, S., TORRI, V., VALSECCHI, M.G., *et al.* (1990). Prognostic factors in advanced epithelial ovarian cancer. *Br. J. Cancer*, **62**, 444–450.

MCGUIRE, W.L. (1991). Breast cancer prognostic factors: evaluation guidelines. *J. Natl Cancer Inst.*, **83**, 154–155.

PETO, R., PIKE, M.C., ARMITAGE, P., *et al.* (1977). Design and analysis of randomised clinical trials requiring prolonged observations of each patient. II Analysis and examples. *Br. J. Cancer*, **35**, 1–39.

REDMAN, C.W.E., BLACKLEDGE, G.R., KELLY, K., POWELL, J., BUXTON, E.J. & LUESLEY, D.M. (1990). Can early serum CA125 response predict outcome in epithelial ovarian cancer? *Eur. J. Cancer*, **26**, 593–596.

REES, G.J.G. (1990). Cancer treatment: deciding what we can afford. *Br. Med. J.*, **302**, 799–800.

ROSEN, A., SEVELDA, P., KLEIN, M., SPONA, J. & BECK, A. (1990). A score as a prognostic index in patients with ovarian cancer. *Arch. Gynecol. Obstet.*, **247**, 125–129.

RUSTIN, G.J.S., GENNINGS, J.N., NELSTROP, A.E., COVARRUBIAS, H., LAMBERT, H.E. & BAGSHAWE, K.D. (1989). Use of CA125 to predict survival of patients with ovarian carcinoma. *J. Clin. Oncol.*, **7**, 1667–1671.

RUSTIN, G.J.S. (1991). Impact of tumour marker measurements upon management of patients with carcinomas of the ovary. *Dis. Markers*, **9**, 153–158.

SAS/STAT USERS GUIDE. (1990). The Lifetest Procedure. Version 6, 4th Ed., Volume 2, SAS Inst. Inc. Cary NC, pp 1027–1069.

SCHRAY, M., MARTINEX, A., COX, R. & BALLON, S. (1983). Radiotherapy in epithelial ovarian cancer. Analysis of prognostic factors based on long term experience. *Obstet. Gynecol.*, **62**, 373–382.

SEVELDA, P., SCHEMPER, M. & SPONA, J. (1989). CA125 as an independent prognostic factor for survival in patients with epithelial ovarian cancer. *Am. J. Obstet. Gynecol.*, **161**, 1213–1216.

SWENERTON, K.D., HISLOP, T.G., SPINELLI, J., LE RICHE, J.C., YANG, N. & BOYES, D.A. (1985). Ovarian carcinoma: a multivariate analysis of prognostic factors. *Obstet. Gynecol.*, **65**, 264–270.

THIGPEN, T., BLESSING, J., BALL, H., HUMMEL, S. & BARRET, R. (1990). Phase II trial of taxol as second-line therapy for ovarian carcinoma: a Gynecologic Oncology Group Study. *Proc. Am. Soc. Clin. Oncol.*, **9**, 156 (Abstract 604).

VAN DER BURG, M.E.L., LAMMES, F.B., VAN PUTTEN, W.L.J. & STOTER, G. (1988). Ovarian cancer: the prognostic value of the serum half-life of CA125 during induction chemotherapy. *Gynecol. Oncol.*, **30**, 307–312.

VAN DER ZEE, A.G.J., DUK, J.M., AADLERS, J.G., BOONTJE, A.H., HOOR, K.A.T. & DE BRUIJN, H.W.A. (1990). The effect of abdominal surgery on the serum concentration of the tumour-associated antigen CA125. *Br. J. Obstet. Gynaecol.*, **97**, 934–938.

VERGOTE, I.B., BORMEN, O.P. & ABELER, V.M. (1987). Elevation of serum CA125 levels in the monitoring of ovarian cancer. *Am. J. Obstet. Gynecol.*, **157**, 88–92.

WEBB, M.J. (1989). Cytoreduction in epithelial ovarian cancer: achievability and results. *Ballière's Obstet. Gynaecol.*, **3**, 83–94.

WILLIAMS, C. (1992). Implications of an overview of chemotherapy in advanced ovarian carcinoma. *Br. J. Cancer*, **66**, 225–226.